# 肺上沟瘤的治疗进展

**DOI:** 10.3779/j.issn.1009-3419.2018.06.10

**Published:** 2018-06-20

**Affiliations:** 100021 北京，北京协和医学院，国家癌症中心，中国医学科学院肿瘤医院胸外科 Department of Thoracic Surgery, National Cancer Center, Cancer Hospital, Chinese Academy of Medical Sciences & Peking Union Medical College, Beijing 100021, China

**Keywords:** 肺上沟瘤, 同期放化疗, 手术治疗, Pancoast tumor, Chemoradiotherapy, Surgical resections

## Abstract

肺上沟瘤是指发生在肺上沟区的的支气管源性肿瘤, 是非小细胞肺癌(non-small cell lung cancer, NSCLC)的一个独特的临床亚型, 占肺癌总数不足5%。它常侵犯第1肋、臂丛、锁骨下动静脉、交感神经链、星状神经节和(或)椎体等胸廓入口结构。近几十年, 肺上沟瘤的治疗取得了不断的进展, 最新发布的几个临床试验证实了同期放化疗加手术切除能够改善肿瘤的完整切除率、局部控制率和病理缓解率, 延长患者的总生存时间。已经成为肺上沟瘤的治疗最为有效的方式, 并成为美国国立综合癌症网络(National Comprehensive Cancer Network, NCCN)和美国胸科医师协会(American College of Chest Physicians, ACCP)指南推荐的肺上沟瘤治疗方案。本文回顾国内外相关文献, 简要介绍肺上沟瘤手术治疗及综合治疗的进展情况。

## Abstract (en)

Superior sulcus tumor of the lung is a bronchogenic tumor occurred in the apex of the upper lobe of the lung and is a unique clinical subtype of non-small cell lung cancer (NSCLC), which account for less than 5% of all bronchogenic carcinomas.It often involves the first rib, brachial plexus, subclavian vessels, sympathetic chain, stellate ganglion or vertebra.A lot of progress has been achieved in the treatment of superior sulcus tumor over the past decades.Several clinical trials reported in recent years have confirmed that concurrent chemoradiotherapy followed by surgical resection can improve the rate of complete resection, local control and pathological remission of the tumor, and prolong the total-survival time.It has become the most effective treatment mode for the superior sulcus tumor, and recommended as a standard treatment mode for superior sulcus tumor by National Comprehensive Cancer Network (NCCN) and American College of Chest Physicians (ACCP) guidelines.This article reviews relevant literatures at home and abroad, and briefly introduces the advances in surgical treatment and comprehensive treatment of superior sulcus tumor.

肺上沟瘤是指发生在肺上沟区的支气管源性肿瘤, 是非小细胞肺癌(non-small cell lung cancer, NSCLC)的一个独特的临床亚型, 占肺癌总数不足5%^[1]^。它常侵犯第1肋、臂丛、锁骨下动静脉、交感神经链、星状神经节和(或)椎体等胸廓入口结构。来自费城的放射学家Pancost HK第一个详细描述了这类特殊的肿瘤, 故也称Pancoast瘤。1946年Herber和Watson^[2]^随访了8例肺上沟瘤患者, 发现所有的患者在确诊后10个月内死亡。在1950年以前, 肺上沟瘤被认为是无法切除的。1954年Haas等^[3]^对肺上沟瘤患者采取姑息性外照射, 结果4例患者疼痛得到了明显缓解。1956年, Chardask和MacCallum^[4]^首先尝试着对1例患者采取了外照射加手术切除, 该患者存活时间超过了5年。Shaw和Paulson^[5]^对18例患者采取局部放疗30 Gy后再后路行根治性手术, 较以往的治疗方式获得更高的5年生存率和局部控制率。此后, 这种治疗方案得到迅速推广和改进, 并作为肺上沟瘤的标准治疗模式沿用数十年。在过去的几十年间, 各种新技术不断被开发应用, 为肺上沟瘤的切除提供了更加安全有效的方法, 在处理那些侵犯锁骨下血管、臂丛神经、椎体等结构的肿瘤时, 安全性得以提高。最近20年, 随着外科技术的进步和同期放化疗的应用, 肺上沟瘤的治疗效果有了较大进步。同期放化疗加手术切除被证实能够提高肿瘤的完整切除率和局部控制率, 延长患者的总生存时间, 成为最有效的肺上沟瘤治疗模式。现对肺上沟瘤的治疗研究进展做一综述及解读。

## 1 外科治疗的进展

### 1.1 后胸入路术式

高后外侧胸廓切口(Paulson-Shaw术式)是肺上沟瘤的经典术式^[6]^。这一术式的优点在于能充分暴露后侧胸壁、胸廓入口中后部以及肺门, 尤其显露脊椎、胸神经根较理想, 便于进行受累脊椎和胸神经根的切除。缺点是对胸顶壁、前壁的暴露很差, 难以探查肿瘤对锁骨下血管、胸导管等结构的侵犯程度及进行手术切除。Niwa等^[7]^设计了一种钩形切口, 它包绕上述切缘并向上前方斜行至胸锁关节, 与Paulson-Shaw术式相比, 它能够翻起肩胛骨从而更好暴露胸壁及前胸壁顶部结构, 完成受侵锁骨下血管、同侧锁骨上淋巴结、臂丛及椎体的切除。Masoka术式的缺点是切口较大, 肩胛带肌肉破坏广泛, 容易导致术后严重的切口并发症, 如切口不愈合、术后上肢活动受限等, 因此未得到广泛推广。上三肋及肩胛下的胸壁的缺损一般不需要修补, 肩胛骨和锁骨可以遮盖第1-3肋骨骨性缺损。Marra等^[8]^报道至多40%的病例需要胸壁重建。

### 1.2 前胸入路术式

Dartevelle等^[9]^于1993年提出了经颈胸前路切口(anterior transcervical-thoracic approach), 对于提高肺上沟瘤手术切除的安全性和肿瘤的完整切除率有较大帮助。其最大优点是可以充分显露胸廓入口重要组织结构, 经此切口可直接探查肿瘤胸廓入口如锁骨下血管、臂丛、胸导管等结构的侵犯程度, 同时可以彻底、安全地游离、切除受累的锁骨下血管和部分臂丛神经, 方便进行锁骨下血管的重建。此外, 对于椎体前部受侵的患者可经该切口行半椎体切除。因此对于靠前的肺尖部肿瘤, 尤其是侵及锁骨下血管时, 经该切口可方便地切除肿瘤。该术式的缺点在于对于后胸壁、肺门血管显露差, 通常还需要再行后外侧切口来协助完成肺叶切除术。Masaoka等^[10]^报道采用trans-sternal切口, Bains等^[11]^和Rusca等^[12]^采取Hemiclamshell切口进行肺上沟瘤的手术治疗, 上述两种切口保留了胸锁关节的完整性, 克服了经典Dartevelle术式的缺点, 便于进行肺叶切除、淋巴结清扫、椎体的显露, 并且不破坏胸锁关节, 术后无明显畸形。该术式的缺点是术后可能发生胸骨不愈合及连枷胸。此外, 近年来有学者尝试前路联合后路入式进行肺上沟瘤切除术, 并被证实手术安全、有效。

### 1.3 有椎体侵犯的肿瘤切除

术前同步放化疗和椎体内固定技术的发展, 使得许多有椎体侵犯的T4期肿瘤切除变得可能。Domeester等^[13]^首先尝试联合椎体切除治疗侵犯脊柱的肺癌, 取得较好的效果。Fadel等^[14]^报道的5年生存率为20%;而Walsh等^[15]^报道胸壁肿瘤侵犯椎体的外科5年生存率更可达50%。Gokaslan等^[16]^回顾了自1985-1999年间42例行椎体切除的患者, 结果完整切除的中位生存期为2.8年, 不完整切除的中位生存期仅为0.8年。此外, Deutsch^[17]^和Walsh等^[18]^报道椎体切除及椎管减压术能明显改善因椎体破坏和脊髓压迫所致疼痛及不全瘫痪, 有效率达80%-90%, 并认为脊柱稳定对于缓解疼痛和神经压迫有更明显的作用。因此对于有手术指征的肺上沟瘤伴椎体侵犯患者应尽早手术, 以期彻底切除原发肿瘤病灶及受侵椎体, 最终达到根除肿瘤、迅速缓解疼痛、提高患者生活质量的目的。

### 1.4 电视胸腔镜辅助技术在肺上沟瘤治疗中的临床应用

肺上沟瘤的外科手术具有挑战性, 胸廓入口特殊的解剖结构给手术带来了极大的难度, 手术时除了需要进行上肺叶切除及淋巴结清扫以外, 还需对受累胸壁行整块根治性切除(radical *en-bloc* resection), 手术最困难的地方在于术野的显露和胸腔粘连的松解。随着微创胸部外科的发展, 电视胸腔镜技术(video-assisted thoracic surgery, VATS)被广泛应用于早期NSCLC的手术治疗之中, 技术已经较为成熟。近年来, 国内外有些学者开始将电视胸腔镜技术运用于肺上沟瘤的诊断及手术治疗。最初有些学者将其运用于手术开始阶段的胸腔探查, 以探明胸腔内肿瘤对胸壁、胸廓入口、肺以及纵隔的侵犯情况^[19]^。Truin等^[20]^于2010年将胸腔镜应用于肺上沟瘤手术中进行肺叶切除, Koshiko等^[21]^及Linden等^[22]^各有1例前径路切除Pancoast瘤术中采用VATS行肺叶切除术的报道。在Caronia等^[23]^的研究中, 他们报道了一组来自4个不同肿瘤中心的7例肺上沟瘤病例, 所有患者均在胸腔镜下联合前/后入路完成肺叶及受累胸壁的完整切除、淋巴结清扫。国内Jiao等^[24]^报道6例电视胸腔镜联合前/后径路完成肺上沟瘤切除术病例, 探索了手术程序的优化。Kawai等^[25]^尝试了胸壁分割小切口胸腔镜辅助治疗肺上沟瘤, 并证实较传统开胸手术术野显露更好、创伤小、出血量少等优点。他们的初步经验显示应用胸腔镜辅助行肺上沟瘤切除术是安全、可靠的。并且可以减少骨性胸廓的切除范围, 减少开胸手术的并发症, 减轻患者痛苦, 促进术后快速康复。目前的不足之处在于报道病例数较少, 并且缺乏对照研究。今后应联合多中心完成胸腔镜对照传统开胸手术治疗肺上沟瘤的前瞻性临床对照研究, 则能够进一步明确胸腔镜在肺上沟瘤手术治疗中的应用价值。

## 2 综合治疗进展

肺上沟瘤的治疗经历了单纯放疗, 放疗+手术治疗、同期放化疗+手术治疗的不断发展。20世纪50年代起术前放疗+手术治疗开始应用于肺上沟瘤的治疗, 此后几十年, 一直被认为是肺上沟瘤的标准治疗方案。最初, 所有接受术前放疗的患者手术完整切除率只有60%, 5年生存率只有30%。20世纪90年代起, 同步放化疗联合手术治疗开始应用于肺上沟瘤的治疗, 来自美国西南肿瘤协作组的试验(SWOG9416)^[26]^证实了这种治疗模式的有效性。该试验纳入了1995年4月-1999年11月110例肺上沟瘤患者, 所有患者均经胸腔镜证实无纵隔淋巴结转移(T3-4N0-1M0), 有104例(95%)患者完成了诱导治疗, 88例(80%)完成了开胸手术, 91%的T3期患者和87%的T4期患者完成了R0切除, 其中61例(56%)达到了病理完全缓解。患者5年总体生存率为44%, R0切除患者5年生存率为54%, T3期和T4期肿瘤未见明显差异。Waseda等^[27]^研究结果也显示T3期和T4期患者在5年生存率和5年无病生存率方面无显著差异。该试验纳入了46例1998年-2013年行同步放化疗联合手术治疗的患者, 其中28例(61%)为T4期, 这是迄今为止所有临床试验中T4期肿瘤占比最高的。该研究显示T3期和T4期肿瘤患者在5年生存率和5年无病生存率方面无显著差异, 这提示T4期肿瘤通过行扩大的肿瘤切除术可以获得与T3期肿瘤相似的获益, 当然这需要高超的手术技巧和多学科团队的共同合作。

日本临床肿瘤协作组的试验(JCOG9806)^[28]^使用了和SWOG9416相同的治疗方案, 5年总体生存率为56%。在这两个临床试验中, 威胁患者长期生存最主要因素是远处转移, 约占复发患者的80%。为降低术后远处转移发生率, SWOG9416对接受手术的患者术后增加了2个周期的巩固化疗(顺铂+依托泊苷), 但并未取得满意效果。美国西南肿瘤协作组对以顺铂为基础的行术前同期放化疗+手术治疗的Ⅲb期NSCLC患者采用多西他赛进行术后巩固治疗, 结果显示这可能是一种有效的治疗方案^[29]^。为研究多西他赛的有效性, 美国西南肿瘤协作组进行了新的临床试验(SWOG 0200)^[30]^, 该试验纳入了44例T3-4N0-1M0肺上沟瘤患者, 其中29例(66%)进行了手术切除, 22例患者接受了术后巩固化疗, 其中20例(90%)完成了3个周期的化疗。最新随访结果已经显示出患者从该治疗中获益, 中位生存期是45个月, 中位无进展生存期43个月, 平均总生存期50个月, 3年无进展生存率56%, 3年总生存率61%。

肺上沟瘤采用术前放化疗加手术治疗的方法至今已20余年, 2014年Toyokawa等^[31]^回顾了这些研究结果。纳入的都是Ⅱ期临床试验, 包括2个单中心和3个多中心试验。近75%的病例是T3期, 剩下的为T4期, 70%-90%为N0。诱导同步放化疗结束后手术可切除率70%-80%, 其中Marra试验可手术切除率达到94%。在病理完全缓解率方面, SWOG9416和SWOG0200为28%-36%, Marra试验为45%, Kunitoh试验为21%。SWOG9416和SWOG0200进行了术后巩固治疗。SWOG试验中位生存期为3年-4年。在这些试验中, 完全切除或近完全切除的患者获得了明显的生存获益及较好的局部控制率。影响患者长期生存的主要因素是远处转移, 这些远处转移多发生在术后2年-3年, 最常见转移部位为脑。有人主张对于R0切除的患者进行预防性颅脑照射, 但其效果目前仍不确切, 需要进一步研究证实^[32]^。

## 3 靶向治疗在肺上沟瘤治疗中的价值

研究^[33]^显示有19号外显子碱基对缺失或21号外显子突变的肺腺癌对于表皮生长因子受体(epidermal growth factor receptor, EGFR)酪氨酸激酶抑制剂高度敏感, 表现出高反应率和无进展生存率。*EGFR*基因突变的存在是EGFR受体抑制剂治疗肺腺癌良好预后的强有力预测因子, 因此EGFR受体抑制剂如吉非替尼在肺腺癌的初始治疗及远处转移的控制方面可能优于卡铂+紫杉醇联合化疗方案。超过50%的肺尖部的肿瘤是肺腺癌, 其余主要为鳞癌和大细胞癌^[34-36]^, 在这个位置小细胞癌所占比例极小^[37]^。同时, 肺上沟瘤和其他部位的肺癌具有相同的生物学行为, 遵循相同的治疗原则^[34]^。这把肺上沟瘤和表皮生长因子受体联系在了一起, 这表明肺上沟瘤患者可能会从靶向治疗中获益, 但这仍需进一步前瞻性临床随机对照研究证实。

## 4 小结

在过去的几十年中, 肺上沟瘤的治疗取得了显著的进展。其中在外科手术方面最大的成就是前胸入路的引入和不断改进, 这为以前被认为不能进行手术的臂丛、锁骨下血管受侵的肺上沟瘤患者带来了治愈的可能。胸腔镜技术在肺上沟瘤治疗中的价值初露端倪, 它具有术中视野好、创伤性小、术后恢复快等优点, 但它对于临床医生的手术操作技能要求也较高, 目前仍需要进一步的临床前瞻性对照研究证实其在肺上沟瘤治疗中的应用价值。手术入路应根据肿瘤的位置及肿瘤侵犯的胸廓入口结构进行个体化选择, 以期尽可能达到R0切除。

诸多临床试验证实同期放化疗联合手术治疗在改善病人长期生存率、局部控制率、肿瘤R0切除率方面取得了较为满意的效果。目前这种治疗策略已经被广泛接受, 并成为NCCN和ACCP指南推荐的肺上沟瘤治疗方案。N2-3淋巴结转移及远处转移等晚期患者的治疗仍是肺上沟瘤治疗的难点, 随机分组研究显示NSCLC根治性切除术后行系统性化疗可以降低远处转移的发生率, 并有5%-15%的5年生存率获益^[38]^。这也是未来肺上沟瘤治疗的研究方向之一。

总之, 肺上沟瘤的治疗方案需要根据肿瘤分期及患者自身条件进行选择个体化的综合治疗, 以获得最理想的治疗效果。
